# All-trans-retinoic acid ameliorates atherosclerosis, promotes perivascular adipose tissue browning, and increases adiponectin production in Apo-E mice

**DOI:** 10.1038/s41598-021-83939-x

**Published:** 2021-02-24

**Authors:** Małgorzata Kalisz, Magdalena Chmielowska, Lidia Martyńska, Anita Domańska, Wojciech Bik, Anna Litwiniuk

**Affiliations:** 1 Department of Neuroendocrinology, Centre of Postgraduate Medical Education, Marymoncka 99/103, 01-813 Warsaw, Poland; 2 Department of Physiological Sciences, Institute of Veterinary Medicine, Warsaw University of Life Sciences - SGGW, Nowoursynowska 159, 02-776 Warsaw, Poland

**Keywords:** Drug discovery, Physiology, Cardiology, Diseases

## Abstract

All-trans-retinoic acid (atRA), an active metabolite of vitamin A, exerts a potential role in the prevention of cardiovascular diseases. It has been shown that atRA ameliorates atherosclerosis while the exact mechanism underlying this protection remains unknown. This study investigated the influence of atRA on insulin resistance (IR), atherosclerosis, and the process of perivascular adipose tissue (PVAT) browning. Moreover, syntheses of adiponectin, adipokine with anti-atherogenic effects, and tumor necrosis factor-alpha (TNF-α), a pro-inflammatory cytokine, were determined in PVAT. Apolipoprotein E-deficient mice (Apo-E) and control C57BL/6J wild-type mice were treated with atRA (5 mg/kg/day) or vehicle (corn oil) by plastic feeding tubes for 8 weeks. Long-term atRA treatment in Apo-E mice did not affect insulin resistance. AtRa administration ameliorated atherosclerosis, induced PVAT browning, and increased adiponectin production in PVAT in Apo-E mice. Furthermore, atRA increased nitric oxide (NO) level but did not affect adiponectin concentration in the aorta of Apo-E mice. These results indicate that atRA ameliorates atherosclerosis in Apo-E mice. We also observed the browning of PVAT. Besides, atRA increased the synthesis of adiponectin in PVAT and augmented NO level in the aorta in ApoE mice.

## Introduction

The perivascular adipose tissue (PVAT) has a mixed composition of both types of white (WAT) and brown adipose tissue (BAT)^1^. In mice, the mediastinal depot (coronary artery and thoracic aorta) is composed mainly of BAT, the abdomino-pelvic depot (iliofemoral vessels) equally consists of WAT and BAT, while the retroperitoneal and mesenteric depots (abdominal aorta and mesenteric artery) contain primarily WAT^1^. PVAT is located outside of the blood vessel with direct contact with the adventitial layer. PVAT is recognized as an active endocrine organ that secrets several adipokines playing an important role in vascular function. Among the adipokines, adiponectin is an endogenous insulin-sensitizing hormone, which exerts vasculoprotective and anti-inflammatory effects. It has been demonstrated that adiponectin limits the initiation of atherosclerotic plaque formation^2^. More specifically, adiponectin decreases the mRNA level of class A macrophage scavenger receptors, reduces foam cell formation, and inhibits the secretion of proinflammatory cytokines^3^. Previous in vitro studies demonstrated that adiponectin induces nitric oxide (NO) synthesis by stimulation of endothelial NO synthase (eNOS) production through activation of the AMP-activated protein kinase (AMPK)^4^. Moreover, adiponectin attenuates tumor necrosis factor-alpha (TNF-α)–induced monocyte adhesion to endothelial cells^5^.

Vitamin A is involved in the regulation of cardiovascular risk factors like glucose concentration, lipid metabolism, and inflammation^6^. Two forms of biologically active metabolites of retinoic acid (RA): all-trans-retinoic acid (ATRA) and 9-cis retinoic acid (9-cis RA), regulate a wide variety of physiological functions through two receptors: the RA receptors (RARs), which are activated by both all-trans and 9-cis RA, and the retinoid X receptors (RXRs), which are activated specifically by the 9-cis isomer^7^. Previous studies have revealed the protective effects of atRA on the cardiovascular system^8–10^. It has been demonstrated that atRA inhibits experimental atherosclerosis by antioxidant and anti-inflammatory action and inhibition of platelet function^8^. Moreover, atRA enhances reverse cholesterol transport from macrophages^11^.

RA has been shown to promote the WAT browning^12^ and its administration to mice upregulates the expression of uncoupling protein 1 (UCP1) both in vivo and in vitro^13^. BAT is increasingly recognized as a potential target to reduce atherosclerosis development and has an important role in lipoprotein metabolism as thermogenesis consumes large amounts of fatty acids^14^. Experiments with the use of a mouse as a model demonstrated that activation of BAT by cold temperature enhances the clearance of plasma lipids and prevents the development of atherosclerosis^15,16^. PVAT is a unique adipose tissue depot that has the potential to profoundly perturb the vasculature under certain conditions^17^. Atherosclerotic lesions were significantly increased in mice with impaired PVAT development, thus indicating that the lack of PVAT is sufficient to increase atherosclerosis^18^. In the PVAT surrounding critical plaques, macrophages and lymphocytes numbers were higher than in segments without atherosclerosis^19^. Recently, it has been shown that angiotensin 1–7 mimetic AVE0991 exhibited anti‐inflammatory properties affecting monocyte/macrophage differentiation and recruitment to perivascular space in Apo-E mice^20^. Stimulation of PVAT browning seems to be a promising new therapeutic target in obesity and related complications like atherosclerosis.

In the current study, we used the Apo-E mouse model that develops lesions with morphological similarity to human atherosclerotic plaque^21^. We investigated the role of atRA in atherosclerosis and the process of PVAT browning. Further, we explored the atRA influence on insulin resistance (IR), and pro-inflammatory and anti-inflammatory adipokines synthesis in PVAT. We also measured adiponectin and NO levels in the aorta of the atRA- or vehicle-treated mice.

## Materials and methods

### Materials

Dulbecco's Modified Eagle Medium, and heat-inactivated fetal bovine serum (FBS) were purchased from Gibco Life Technologies (Grand Island, NY, USA). Antibiotic antimycotic solution (Penicillin: Streptomycin: Neomycin solution), all-trans retinoic acid, corn oil, bovine serum albumin, O.C.T. tissue-freezing medium, Oil red O, paraffin, PBS, citrate buffer, hydrogen peroxide, 3,3′ diaminobenzidine, PMSF, Protease Inhibitor Cocktail, and NP-40 were purchased from Sigma-Aldrich Chemical Co. (St. Louis, MO, USA). Glucose concentration was measured using a glucometer (Accu-chek Performa, Roche, Mannheim, Germany). Antibodies to the following targets were used: anti-UCP-1 polyclonal antibody was purchased from Cloud-Clone Corp. ((CCC), USA), AKT mouse monoclonal antibody was purchased from Santa Cruz Biotechnologies (USA) and rabbit anti- IgG–horseradish peroxidase secondary antibodies were purchased from Abcam (Cambridge, UK). Plastics and EDTA/aprotinin vacutainer tubes were from Becton Dickinson (BD Biosciences, Franklin Lakes, NJ, USA).

### Experimental animals

8-week-old males of C57BL/6J Wild-type and Apo-E mice were obtained from Charles River Laboratories (Wilmington, MA, USA). The animals were housed under controlled conditions with 10/14-h light/dark cycles and temperature (22 °C). Mice were fed standard chow providing 25% calories from protein, 8% from fat, and 67% from carbohydrates (Morawski, Poland). Animals were fed ad libitum with free access to water. The animals were divided into four groups: C57BL/6J wild-type (n = 5), C57BL/6J wild-type treated with atRA (n = 6), Apo-E mice (n = 5) and Apo-E mice treated with atRA; (n = 5). AtRA (5 mg/kg/day) or vehicle (corn oil) were administered orally by plastic feeding tubes for 8 weeks. Blood samples were collected after 6 h of fasting. The animals were anesthetized with ketamine and xylazine and then euthanized. Plasma and tissues were collected and stored at − 80 °C until analysis. For quantification of the atherosclerotic lesions in the aortic root, the entire aorta, between the heart and iliac arteries was dissected. The heart with an attached 2–3 mm long aortic arch was dissected and the lower ~ 70% of the ventricular mass was cut away. The upper cardiac portion was frozen in the O.C.T. tissue-freezing medium (Sigma-Aldrich Chemical Co., St. Louis, MO, USA) and stored at − 80 °C with the aortic arch positioned upwards using a metal mold. The abdominal aorta with PVAT between the left renal vein and the iliac bifurcation were isolated under sterile conditions and cut into two portions. In one-part PVAT was isolated from the aorta under a dissecting microscope and both tissues were frozen and stored at − 80 °C until analysis. The second part of aorta and PVAT was fixed in 4% paraformaldehyde (Sigma-Aldrich Chemical Co., St. Louis, MO, USA) until further use.

All animal procedures were performed according to the Polish Guide for the Care and Use of Animals and were approved by the Local Ethics Committee of the Warsaw University of Life Sciences, Poland. All experiments and methods were carried out in compliance with relevant regulations and Animal Research: Reporting of In Vivo Experiments (ARRIVE) guidelines.

### Evaluation of insulin resistance

Plasma samples were collected from the tail vein before and after 8 weeks of treatment with atRA or vehicle. After 6 h of fasting blood samples were collected in EDTA/aprotinin tubes. Glucose concentration was measured using a glucometer (Accu-chek Performa, Roche, Mannheim, Germany). Insulin was determined using a commercially available ELISA kit (Mercodia AB, Uppsala, Sweden), according to the manufacturer’s protocol. The detection limit was ≤ 0.025 μg/L. Intra-assay and inter-assay coefficient of variation were < 3% and < 5%, respectively. The HOMA-IR index was calculated as (fasting serum glucose × fasting serum insulin/22.5) to assess IR^22^.

### Cholesterol quantification

Plasma cholesterol level was quantified with a colorimetric method using the HDL and LDL/VLDL (Abcam, Cambridge, UK) according to the manufacturer’s instructions.

### Evaluation of atherosclerotic lesions

Histological analysis of atherosclerotic plaque development was performed in the aortic root. The isolated hearts were frozen in the O.C.T. tissue-freezing medium (Sigma-Aldrich Chemical Co., St. Louis, MO, USA) and cryosectioned at 5 µm. Sections were stained with the Oil red O and counterstained with hematoxylin. The area of the Oil red O staining in each section was measured for the detection of atherosclerotic lesions.

### PVAT explants

We isolated abdominal PVAT between the left renal vein and the iliac bifurcation by using a dissecting microscope under sterile conditions and prepared tissue culture. PVATs were rinsed in PBS three times and incubated in DMEM media supplemented with 10% fetal bovine serum and 1% (v/v) antibiotic antimycotic solution (Penicillin: Streptomycin: Neomycin solution, 50 IU/mL/50 μg/mL/100 µg/mL; Sigma-Aldrich Chemical Co., St. Louis, MO, USA) in an incubator under standard culture conditions (in a humidified atmosphere of 95% air and 5% CO_2_ at 37 °C). Media and tissue were collected after 24 h. Then, adiponectin and TNF-α protein levels in PVAT and its release into the medium were measured using ELISA. The adiponectin and TNF-α mRNA levels were determined by RT-PCR.

### Real-time quantitative PCR

PVATs were homogenized in a Tissue Lyser (Qiagen, Germany) using a lysis buffer (0.01 M PBS; 0.001 M phenylmethylsulfonyl fluoride (PMSF); Protease Inhibitor Cocktail (Sigma-Aldrich Chemical Co., St. Louis, MO, USA); 1% NP-40 (v/v). Total RNA was isolated with the RNeasy Lipid Tissue Mini Kit (Qiagen, Germany) according to the manufacturer’s protocol. cDNA synthesis was performed using SOLIScript RT Kit (Solis BioDyne, Tartu, Estonia), following the manufacturer’s instructions. Relative mRNA levels were determined by quantitative RT-PCR using 5× HOT FIREPol Eva Green qPCR Mix Plus (Solis BioDyne, Tartu, Estonia) and CFX Connect (Bio-Rad, Hercules, California, USA). Gene expression was normalized to the expression of the *Gapdh* housekeeping gene. Primers are listed in Table 1. The formula 2^−ΔΔCt^ was used to calculate relative gene expression.

**Table 1:** Gene primer sequences.

Gene name	Forward primer	Reverse primer
*Gapdh*	AGACAGCCGCATCTTCTTGT	CTTGCCGTGGGTAGAGTCAT
*Adiponectin*	GATGGCAGAGATGGCACTCC	CTTGCCAGTGCTGCCGTCAT
*Ucp-1*	GGGCCCTTGTAAACAACAAA	GTCGGTCCTTCCTTGGTGTA
*Cidea*	ATCACAACTGGCCTGGTTACG	TACTACCCGGTGTCCATTTCT
*Prdm16*	CAGCACGGTGAAGCCATTC	GCGTGCATCCGCTTGTG

### Western blot analysis

Proteins were extracted from PVAT homogenized in a Tissue Lyser (Qiagen, Germany) using a lysis buffer (0.01 M PBS; 0.001 M phenylmethylsulfonyl fluoride (PMSF); Protease Inhibitor Cocktail (Sigma-Aldrich Chemical Co., St. Louis, MO, USA); 1% NP-40 (v/v). The total protein concentration was determined by the Pierce BCA protein assay kit (Thermo Fisher Scientific, Waltham, USA). Adipose tissue explants lysate protein was separated by the SDS-PAGE and transferred onto polyvinylidene difluoride (PVDF) membrane. The membrane was immunoblotted with an anti-UCP-1 polyclonal antibody (Cloud-Clone Corp. (CCC), USA) and AKT mouse monoclonal antibody (Santa Cruz Biotechnologies, USA) overnight followed by rabbit anti- IgG–horseradish peroxidase secondary antibodies (Abcam, Cambridge, UK). Protein bands were detected with a light-emitting nonradioactive method using ECL reagent prepared in the laboratory^23^. The membranes were then subjected to autoluminography for 1 to 5 min.

### Adiponectin, TNF-α, and NO measurement

PVAT and aorta were homogenized in a Tissue Lyser (Qiagen, Germany) using a lysis buffer (0.01 M PBS; 0.001 M phenylmethylsulfonyl fluoride (PMSF); Protease Inhibitor Cocktail (Sigma-Aldrich Chemical Co., St. Louis, MO, USA); 1% NP-40 (v/v). The concentrations of adiponectin, TNF-α, and NO in PVAT, aorta, and medium were determined using Mouse Adiponectin/Acrp30 Quantikine ELISA Kit, Mouse TNF-alpha DuoSet ELISA Kit and Total Nitric Oxide and Nitrate/Nitrite Parameter Assay Kit (R&D Systems, Minneapolis, MN, USA). The sensitivity of adiponectin, TNF-α and NO by ELISA were < 0.007 ng/mL, < 7.21 pg/mL and < 0.78 μmol/L, respectively. Intra-assay and inter-assay coefficient of variation were < 6.7% and < 6.4% for adiponectin assay, < 3.8% and < 7.7% for TNF-α assessment, and < 2.5% and < 4.6% for NO assay, according to the manufacturer.

### Immunohistochemistry

The abdominal aortas from sacrificed animals were fixed in 4% paraformaldehyde for 12 h at 4 °C, then paraffin-embedded and sectioned at 5 µm. Sections of aortic tissues were deparaffinized, rehydrated, and washed with distilled water, 3% hydrogen peroxide to inactivate endogenous staining. Subsequently, sections were heated in 10 mM of citrate buffer (pH, 6.0), blocked with goat serum, and incubated sequentially with the primary antibody against UCP-1 (Cloud-Clone Corp. (CCC), USA) overnight at 4 °C and secondary antibody anti-rabbit (Abcam, Cambridge, UK) for 1 h at RT, respectively. Finally, the samples were incubated with 3,3′ diaminobenzidine (DAB) for coloration and counterstained with hematoxylin to stain the nuclei bluish. The positively stained cells showed a brown color. Photographs were taken using the Image-Pro Plus 5.1.

### Statistical analysis

All the statistical analyses were performed using the GraphPad PrismTM version 5.0 software (GraphPad Software Inc., San Diego, CA, USA). All data are presented as mean ± standard error of the mean (SEM).

Multiple comparisons were performed using a one-way analysis of variance (ANOVA) with appropriate post hoc analyses. Testing the significance of atRA on glucose and insulin levels before and after experiments were performed with two-way ANOVA. Values of p < 0.05 were considered statistically significant.

## Results

### The influence of the all-trans-retinoic acid treatment on insulin resistance

There was no difference in body weight between the vehicle-treated and the atRA-treated groups of Apo-E mice (Table 2). Similarly, there were no differences in body weight between subgroups of C57BL/6J mice the vehicle vs. atRA treated groups, respectively (Table 2). Body weight was higher in the vehicle and atRA-treated Apo-E mice when compared to C57BL/6J mice (Table 2).

**Table 2:** Body weight and HOMA-IR before and after 8 weeks of atRA or vehicle treatment.

	Before 8 weeks atRA or vehicle administration		After 8 weeks atRA or vehicle administration	
	Body weight (g)	HOMA-IR	Body weight (g)	HOMA-IR
C57BL/6J vehicle	21.20 ± 0.61	1.13 ± 0.04	24.86 ± 1.08	0.76 ± 0.23
C57BL/6J atRA	20.93 ± 0.82	1.14 ± 0.02	24.08 ± 1.15	0.9 ± 0.22
Apo-E vehicle	24.92 ± 1.94***	0.95 ± 0.29	27.9 ± 1.46***	0.83 ± 0.25
Apo-E atRA	25.63 ± 0.76***	1.1 ± 0.06	28.92 ± 1.43***	1.39 ± 0.69

Results are presented as means ± S.E.M. of 5–6 mice per group. The results were analyzed by two‐way ANOVA with Bonferroni post‐test.

***p < 0.01 C57BL/6J atRA vs. ApoE atRA; and C57BL/6J vehicle vs. ApoE vehicle.

To define the influence of atRA on IR, glucose and insulin levels were measured in Apo-E and C57BL/6J mice before and after 8 weeks of treatment with atRA or vehicle and HOMA-IR (homeostasis model assessment of insulin resistance) was calculated (Table 2). There was no significant difference in HOMA-IR between the vehicle-treated and the atRA-treated group of Apo-E mice, as well as between the vehicle group and the atRA group of C57BL/6J mice (Table 2). No significant differences were observed in Apo-E and C57BL/6J mice HOMA-IR before and after atRA administration (Table 2).

### The influence of the all-trans-retinoic acid treatment on cholesterol level

**Figure 1:**
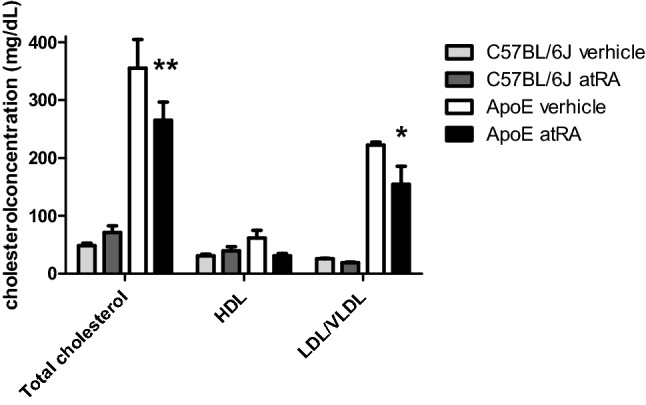
Total cholesterol, HDL, and LDL/VLDL concentrations of C57BL/6 J and Apo-E mice. All-trans-retinoic acid (5 mg/kg/day) or vehicle (corn oil) was administered orally by plastic feeding tubes for 8 weeks. Results are presented as means ± S.E.M. of 5–6 mice per group. The results were analyzed by two‐way ANOVA with Bonferroni post‐test. *p < 0.01 ApoE atRA vs. ApoE vehicle.

AtRA decreases the total and LDL/VLDL cholesterol level in Apo-E mice compared with vehicle-treated animals (Fig. 1). There was no significant difference in HDL fraction between those two groups. No significant differences were observed in total cholesterol and its fractions between AtRA- and vehicle-treated C57BL/6 J mice (Fig. 1).

### Assessment of atherosclerotic lesion area

**Figure 2:**
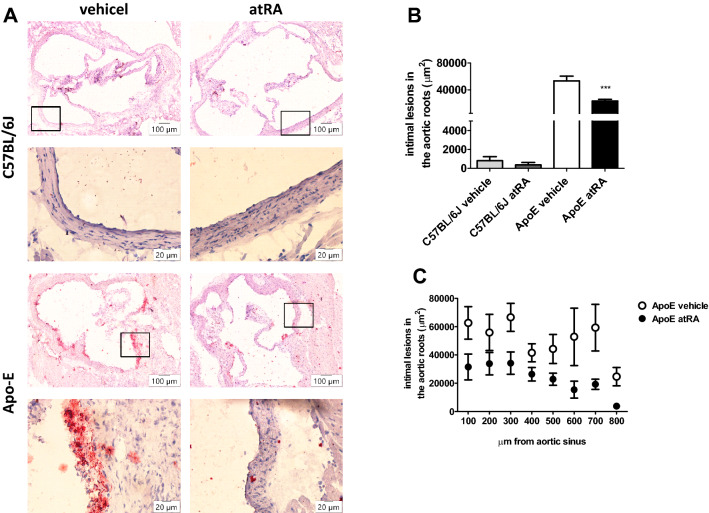
Microscopic analysis of the intimal lesions in the aortic roots. All-trans-retinoic acid (5 mg/kg/day) or vehicle (corn oil) were administered orally by plastic feeding tubes to Apo-E and C57BL/6J mice for 8 weeks. (**A**) The Oil Red O‑stained cross-sections of the aortic roots revealing the area of the lesions (magnification × 10 and × 40). (**B**) Average size of the atherosclerotic plaques in the aortic root. (**C**) Average sizes of the atherosclerotic lesion areas of eight individual cross-sections of the aortic root of each mouse. Data are expressed as the mean ± SEM of 5–6 mice per group. The results were analyzed by one‐way ANOVA with Tukey’s Multiple Comparison Test. ***p < 0.001 ApoE atRA vs. ApoE vehicle.

To analyze atRA influence on the size of atherosclerotic plaques, we performed the Oil Red O staining for measurement of atherosclerotic lesions in the aortic root of mice. C57BL/6J mice did not develop atherosclerosis (Fig. 2). The total area of atherosclerotic lesions of the atRA group was 53% lower than those of the controls of Apo-E mice (Fig. 2).

### The all-trans-retinoic acid treatment induces thermogenic gene expression and formation of beige adipocytes of abdominal perivascular adipose tissue

**Figure 3:**
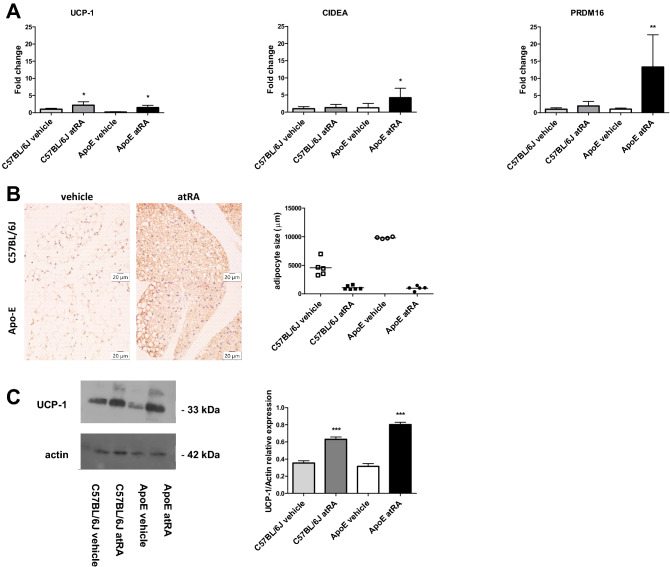
Browning of perivascular adipose tissue after all-trans-retinoic acid administration in Apo-E mice. (**A**) Adipose thermogenic gene expression: *Ucp1*, *Cidea*, and *Prdm16*. (**B**) Perivascular adipose tissue UCP1 immunohistochemical staining. (**C**) UCP1 protein expression was determined by Western Blot. All-trans-retinoic acid (5 mg/kg/day) or vehicle (corn oil) was administered orally by plastic feeding tubes to Apo-E and C57BL/6J mice for 8 weeks. Data are shown as mean ± SEM of 5–6 mice per group. The results were analyzed by one‐way ANOVA with Tukey's Multiple Comparison Test. *p < 0.05 C57BL/6J atRA vs. C57BL/6J vehicle, ApoE atRA vs. ApoE vehicle; **p < 0.01 ApoE atRA vs. ApoE vehicle; ***p < 0.001 C57BL/6J atRA vs. C57BL/6J vehicle, and ApoE atRA vs. ApoE vehicle. Full-length blots are presented in Supplementary Fig. S1.

To investigate whether atRA affects the thermogenic activity of abdominal PVAT, we determined the expression of adipose browning genes and protein level of UCP1. In PVAT of Apo-E mice after 8 weeks of atRA administration, we observed increased expression of the key brown adipose genes: *Ucp1*, *Cidea*, and *Prdm16* (Fig. 3A). We found a significantly higher mRNA level of *Ucp1* in the atRA-treated than in the vehicle-treated C57BL/6J mice (Fig. 3A). Interestingly, there was no significant upregulation of pro-brown adipose tissue gene expression including *Cidea*, and *Prdm16* in the atRA-treated C57BL/6J mice compared to the vehicle-treated C57BL/6J mice (Fig. 3A). In both Apo-E and C57BL/6J mice treated with atRA and in their counterparts, the vehicle-treated Apo-E and C57BL/6J mice, reduced size of adipocytes and induction of formation of beige adipocytes were demonstrated (Fig. 3B). Additionally, western blot analysis was used to determine the effect of the atRA treatment on the UCP1 protein level in PVAT. As shown, the UCP1 level was significantly higher in Apo-E and C57BL/6J mice treated with atRA than in their counterparts, the vehicle-treated Apo-E and C57BL/6J mice, respectively (Fig. 3C).

### The all-trans-retinoic acid treatment increases adiponectin synthesis and secretion by perivascular adipose tissue in Apo-E mice

**Figure 4:**
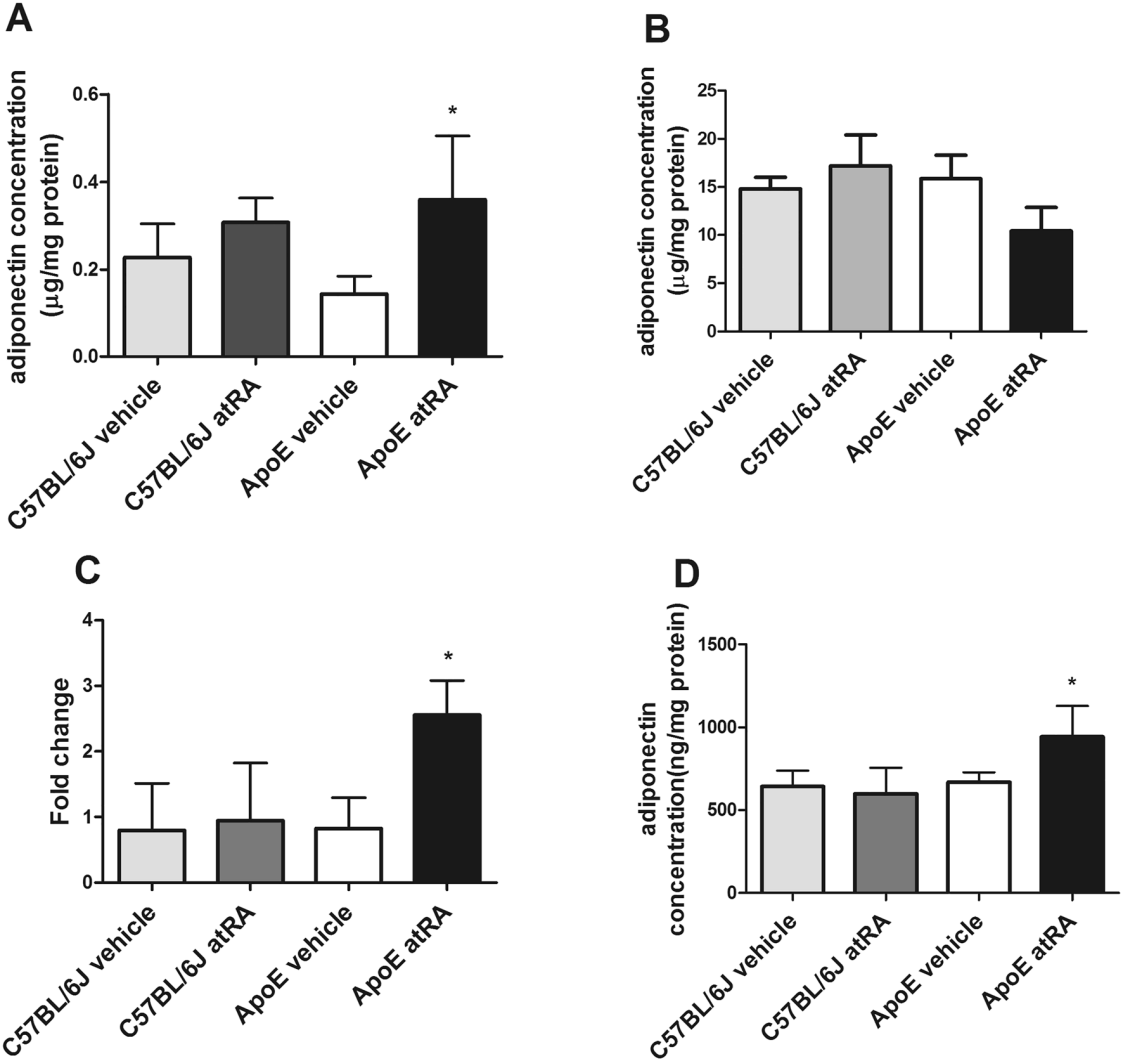
The all-trans-retinoic acid treatment increased adiponectin synthesis and secretion by perivascular adipose tissue. (**A**) Adiponectin concentration in perivascular adipose tissue. (**B**) Adiponectin concentration in visceral adipose tissue. (**C**) mRNA adiponectin level in perivascular adipose tissue. (**D**) Adiponectin concentration in the conditioned medium after 24 h incubation with perivascular adipose tissue explants. All-trans-retinoic acid (5 mg/kg/day) or vehicle (corn oil) were administered orally by plastic feeding tubes to Apo-E and C57BL/6J mice for 8 weeks. Data are shown as mean ± SEM of 5–6 mice per group. The results were analyzed by one-way ANOVA with Tukey’s Multiple Comparison Test. *p < 0.05 ApoE atRa vs. ApoE vehicle.

We tested if the atRA treatment results in altered production of adiponectin, an adipokine with anti-atherosclerotic properties, in PVAT in comparison with adiponectin concentration in visceral adipose tissue. Adiponectin concentration was significantly increased in PVAT of Apo-E group treated with atRA when compared to the vehicle-treated group of Apo-E mice (Fig. 4A). Moreover, there were no significant differences in adiponectin concentrations in visceral adipose tissue in all tested groups (Fig. 4B). After the atRA treatment, adiponectin mRNA level increased in PVAT of Apo-E mice when compared to Apo-E-mice treated with vehicle (Fig. 4C). Additionally, we measured adiponectin secretion to the medium after 24 h incubation from the same PVAT explants. The adiponectin concentration in medium of the atRA-treated Apo-E mice was increased as compared with medium of vehicle-treated Apo-E mice (Fig. 4D). No significant differences in adiponectin mRNA level, protein concentrations, and adiponectin secretion by PVAT were found between the atRA- and the vehicle-treated C57BL/6J mice (Fig. 4).

**Figure 5:**
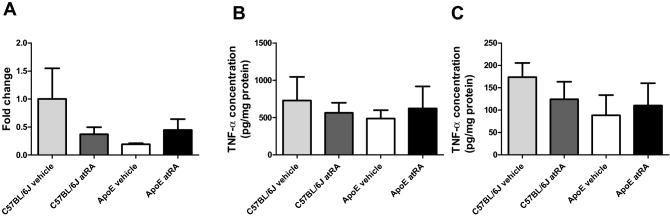
The all-trans-retinoic acid treatment did not affect TNF-α synthesis and secretion by perivascular adipose tissue. (**A**) mRNA TNF-α level in perivascular adipose tissue. (**B**) TNF-α concentration in perivascular adipose tissue. (**C**) TNF-α concentration in the conditioned medium after 24 h incubation with perivascular adipose tissue explants. All-trans-retinoic acid (5 mg/kg/day) or vehicle (corn oil) were administered orally by plastic feeding tubes to Apo-E and C57BL/6J mice for 8 weeks. Data are shown as mean ± SEM of 5–6 mice per group. The results were analyzed by one-way ANOVA with Tukey’s Multiple Comparison Test.

TNF-α is a multifunctional inflammatory cytokine that may play a part in the pathogenesis of atherosclerosis. Therefore, we examined the effect of atRA on the TNF-α concentrations. There was no significant difference in TNF-α mRNA level (Fig. 5A) and protein concentration in PVAT (Fig. 5B) between the atRA- and the vehicle-treated C57BL/6J and Apo-E mice. Moreover, no significant differences of TNF-α were observed in the medium concentrations after 24 h incubation of PVAT explants in all groups of mice (Fig. 5C).

### All-trans-retinoic acid increase NO production in the abdominal aorta of Apo-E mice

**Figure 6:**
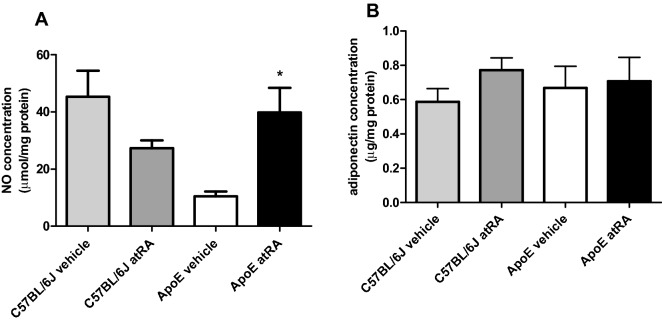
Nitric oxide and adiponectin concentrations in the abdominal aorta of C57BL/6J and Apo-E mice. All-trans-retinoic acid (5 mg/kg/day) or vehicle (corn oil) were administered orally by plastic feeding tubes to Apo-E and C57BL/6J mice for 8 weeks. (**A**) Nitric oxide level in the abdominal aorta. (**B**) Adiponectin concentration in the abdominal aorta. Data are shown as mean ± SEM of 5–6 mice per group. The results were analyzed by one‐way ANOVA with Tukey’s Multiple Comparison Test. *p < 0.05 ApoE atRA vs. ApoE vehicle.

Decreased NO bioavailability, increased O_2_^−^ production, and formation of peroxynitrite have been implicated in the pathogenesis of several cardiovascular diseases including atherosclerosis^24^. We measured NO and adiponectin levels in mice abdominal aorta to test whether atRA can increase their concentrations. We observed increased NO production in the abdominal aorta of the atRA-treated compared to the vehicle-treated Apo-E mice (Fig. 6A). There was no significant difference in NO level in the abdominal aorta between the atRA- and the vehicle-treated C57BL/6J groups (Fig. 6A). Moreover, there was no significant difference in adiponectin concentrations in all groups of mice (Fig. 6B).

## Discussion

Experimental studies point out the beneficial effects of atRA on metabolic health, prevention of atherosclerotic cardiovascular disease (CVD), and browning of WAT^8,12,13^. Results of the present study showed that atRA ameliorates atherosclerosis in Apo-E mice. Moreover, we demonstrated that the atRA treatment stimulated browning of PVAT of the same mouse model. We also observed an increased synthesis and secretion of adiponectin by PVAT. Furthermore, we demonstrated that atRA increased NO concentration in the aorta of Apo-E mice compared to mice treated with vehicle.

Animals are unable to synthesize vitamin A, the major sources for these compounds are formed by forty carbons carotenoids^25^. The key step in the formation of vitamin A (retinol) and its derivatives (retinal, retinoic acid) are oxidative cleavage of b-carotene^25^. The role of vitamin A in glucose and insulin metabolism has not been clearly defined. On one hand, the atRA treatment improved glucose tolerance^26^ and suppressed the expression of leptin^5^ and resistin^20^, adipokines linked to insulin resistance. Moreover, short-term high-dose administration of atRA in high-fat diet mice was associated with a reduction of body weight and an increased UCP-1 expression in WAT^13^. In contrast, in another study, it has been demonstrated that the administration of the RXR agonist did not affect glucose and insulin metabolism^27^. Similarly, in our study, the body weight and HOMA-IR were not significantly different among the groups. The finding of no significant differences in HOMA-IR among the groups suggests unalert insulin sensitivity. We observed an increase in body weight in Apo-E mice compared to C57BL/6J treated with both atRA and vehicle. A recent study has demonstrated a similar effect, Apo-E mice presented an increasing tendency towards weight gain and were heavier compared to their counterparts^28^.

Epidemiological and experimental studies indicate that the antioxidant properties of both β-carotene and vitamin A have the potential to prevent lipoprotein oxidation, which is an initial factor in the development of atherosclerotic CVD^29^. It has been also suggested that AtRA have a potential the same properties^8,29–31^. The protective effects of atRA against atherosclerosis induced by a high-fat diet were demonstrated in the rabbit model, and included inhibited platelet activation and inflammation^8^. This beneficial action was associated with decreased caveolin-1 expression and endothelin-1 secretion, enhanced eNOS activity, and production of NO^8,30,31^. However, the rabbit model does not mimic the human development of aortic lesions because of the different lipoprotein metabolism. Apo-E deficient mice are excellent alternatives to rabbits as models of atherosclerosis in developing lesions with morphological similarity to human atherosclerotic lesions^32,33^. According to data from the literature, the deletion of ApoE is sufficient to drive massive hypercholesterolemia and develop spontaneous lesions under a standard diet, not only on a high-fat diet^34^. Because we used the descending aorta of chow‐fed Apo-E mice before significant atherosclerotic plaque develops, we gained insight into the early phase of the atherosclerosis process. Interestingly, besides dyslipidemia, these mice develop widespread, fibrous plaque-like lesions at the vascular sites.

Previous studies using genetically modified mice revealed novel information on sex dimorphism in the development of cardiovascular phenotype. Mice with Apo-E or low-density lipoprotein receptor deficiency and disruption of estrogen receptors (ER), aromatase or follitropin receptor (both lead to estrogen deficiency) have convincingly shown that estrogen and ER-mediated signaling are protective against vascular dysfunction and atherosclerotic lesions^35–37^. To avoid these hormonal differences, only male mice were used in our experiments.

With the use of the Apo-E model, it has been demonstrated that feeding with β-carotene can inhibit atherosclerosis^38^. In the current study, performed in the same animal model that consumption of a natural β-carotene enriched foods, composed of all-trans and 9-cis isomers, inhibited atherogenesis compared to the vitamin A deficiency diet, while vitamin A did not^39^. Recently, it has been shown that β-carotene and retinoic acid administration reduces the development of atherosclerosis, presumably by a reduction of the secretion of newly synthesized triglyceride and cholesteryl ester^40^. It has also been shown that 9-cis-RA stimulated macrophage cholesterol efflux and inhibited atherosclerotic plaque formation in Apo-E mice^41^. Similarly, in our study, we demonstrated that atRA significantly ameliorated atherosclerosis in the aortic root of Apo-E mice.

According to the literature, the RA administration increased multilocularity, expression of mitochondrial, thermogenic and fatty acid oxidation-related genes as well as retinoblastoma protein (pRb) phosphorylation in mice^13^. Vitamin A deficient diets led to increased body fat and decreased BAT UCP-1 content in mice, and both abnormalities were reversed by the atRA treatment^42^. RA-induced UCP-1 expression in BAT of mice was also demonstrated in other studies^42,43^. UCP-1 expression is regulated by binding specific elements in the UCP-1 enhancer like thyroid hormone receptor (THR), RA receptors (RAR and RXR), and the peroxisome proliferator-activated receptor (PPAR)^43–46^. Our findings confirmed that atRA induced UCP-1 expression in both C57BL/6J and Apo-E mice treated with atRA. However, unexpectedly, we observed increased expression of key brown adipose genes like *Cidea*, and *Prdm-16* only in Apo-E mice not in C57BL/6J. We hypothesized that these differences could be associated with the differences in gene regulation between C57BL/6J and Apo-E mice. PRDM16 is in down-regulated by a miRNA,miRNA-133^47^. Both the corepressor RIP140 and the PPAR coactivator 1α (PGC-1α) play key roles in the regulation of transcription of genes involved in energy homeostasis like CIDEA^48^. To our knowledge, we showed for the first time that the atRA administration induced PVAT browning in Apo-E mice.

It has been suggested that PVAT plays an important role in CVD through its direct local action on the vessels^17^. Herein, we investigated the influence of the atRA administration on browning of abdominal PVAT as it contains primarily WAT^1^. We also examined atRA influence on synthesis and secretion of anti-atherosclerotic and anti-inflammatory adipocytokine adiponectin, and pro-inflammatory TNF-α. The vasodilating effect of adiponectin secreted from PVAT could be achieved by multiple mechanisms. Adiponectin stimulates NO production from endothelial cells and adjacent adipocytes^3,49–51^. Adipose tissue-derived NO may activate large-conductance Ca^2+^-activated K^+^ channels opening in VSMC and leads to vasodilation^52^. The change in NO bioavailability was suggested to be a major mechanism of endothelial dysfunction^53^. NO is an important protective molecule preventing vascular diseases, such as atherosclerosis^53–55^. We demonstrated in Apo-E mice that atRA increased adiponectin synthesis in PVAT. Additionally, high adiponectin concentration was secreted to medium after 24 h incubation of PVAT. We also measured adiponectin concentrations in visceral adipose tissue, but in this type of fat depots, we did not observe any significant differences among all studied groups of mice. Contrary to our results, Zhang et al. observed a decrease of adiponectin mRNA levels in WAT of mice treated with a single dose of atRA^56^. This effect was also confirmed in 3T3-L1 adipocytes^57^. These discrepancies may results from investigation of different depots of adipose tissue as well as different doses and administration time of atRA treatment.

Similarly, no differences were observed in adiponectin concentration in the aorta in all groups of mice. Furthermore, NO level increased in the aorta of the atRA-treated compared to the vehicle-treated Apo-E mice.

It has been demonstrated that adiponectin inhibited TNF-α-induced inflammatory response^4^. Additionally, atRA has been shown to exert immunomodulatory and anti-inflammatory functions in various cell types^58,59^. To our knowledge, so far, the effects of atRA on TNF-α have been investigated in vitro only^60^. It has been revealed that atRA downregulated NO synthase 2 and TNF-α expression, targeting the LPS/TLR4/NF-κB pathway in Colonic Mucosa^60^. In our present study, the atRA treatment did not affect the inflammatory marker, TNF-α, in Apo-E mice.

To sum up, the present study demonstrated that atRA ameliorates atherosclerosis in Apo-E mice. The AtRA treatment in ApoE mice also stimulates browning of PVAT and increases the synthesis of adiponectin. Increased NO concentration in the aorta suggests an important role of PVAT-derived adiponectin in the endothelial function of Apo-E mice.

## Supplementary Information

Supplementary Information.
