# Supplementary material for: All-trans-retinoic acid ameliorates atherosclerosis, promotes perivascular adipose tissue browning, and increases adiponectin production in Apo-E mice
Source: Sci Rep. 2021 Feb 24;11:4451. doi: 10.1038/s41598-021-83939-x (PMC7904836; doi:10.1038/s41598-021-83939-x)

**Supplementary information**

**All-trans-retinoic acid ameliorates atherosclerosis, promotes perivascular adipose tissue browning, and increases adiponectin production in Apo-E mice.**

Małgorzata Kalisz^1^*, Magdalena Chmielowska^1^, Lidia Martyńska^1^, Anita Domańska^1,2^, Wojciech Bik^1^ and Anna Litwiniuk^1^

1 Department of Neuroendocrinology, Centre of Postgraduate Medical Education, Marymoncka 99/103, 01–813, Warsaw, Poland; e-mail: [kzn@cmkp.edu.pl](mailto:kzn@cmkp.edu.pl)

2 Department of Physiological Sciences, Institute of Veterinary Medicine, Warsaw University of Life Sciences - SGGW, Nowoursynowska 159, 02-776 Warsaw, Poland.

* **Corresponding author:** Małgorzata Kalisz; Department of Neuroendocrinology, Centre of Postgraduate Medical Education, Marymoncka 99/103, 01–813, Warsaw, Poland; phone: (22) 569-38-51; e-mail: [mkalisz@cmkp.edu.pl](mailto:mkalisz@cmkp.edu.pl)

**Figure S1.** Full-length blots of Figure 4 UCP-1 and actin in perivascular adipose tissue. Perivascular adipose tissue samples derive from the same experiment and that gels were processed in parallel.


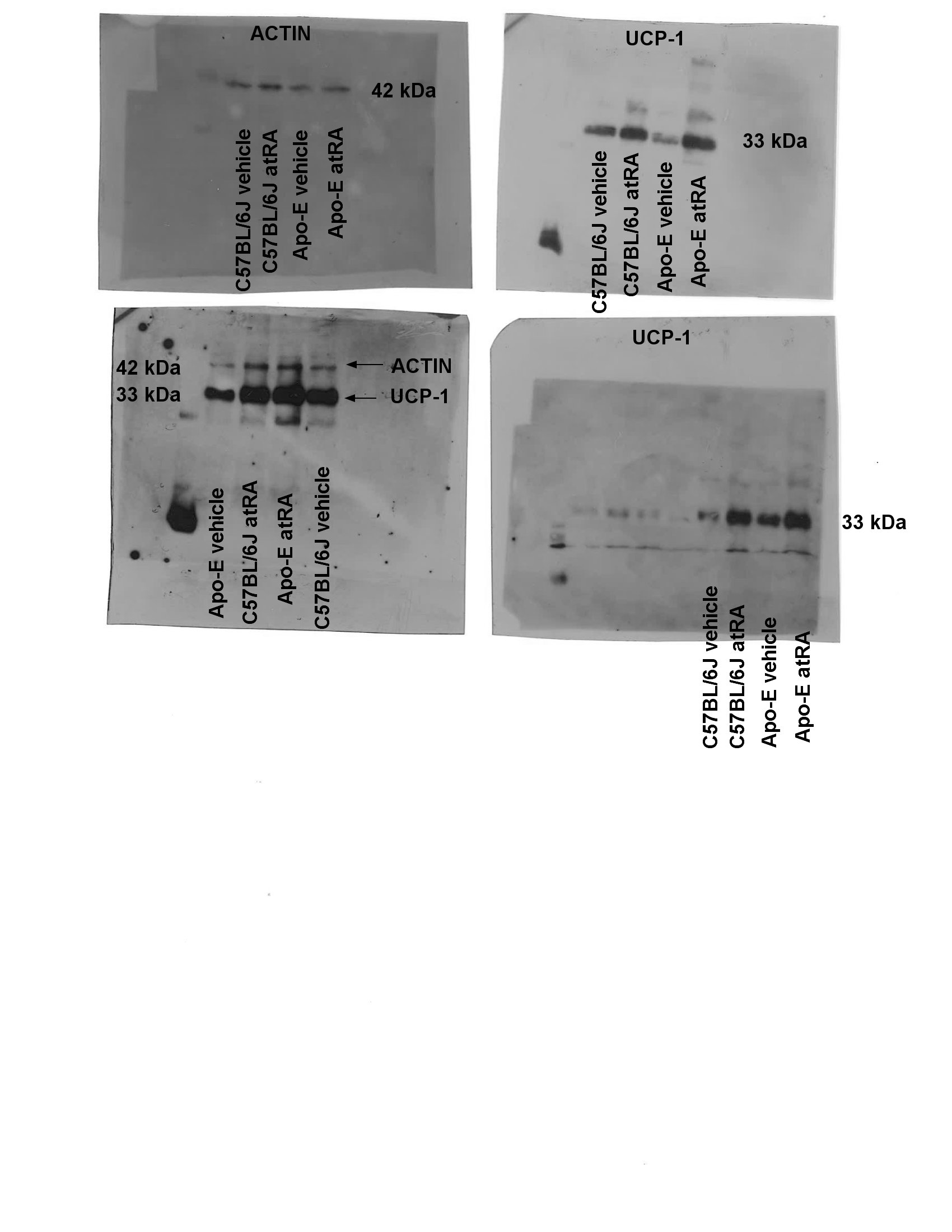

Supplement: Supplementary file 1 — Supplementary Information. [file 41598_2021_83939_MOESM1_ESM.docx]
